# Whole Exome Sequencing Identifies a Novel Hedgehog-Interacting Protein G516R Mutation in Locally Advanced Papillary Thyroid Cancer

**DOI:** 10.3390/ijms19102867

**Published:** 2018-09-21

**Authors:** Woo Kyung Lee, Seul Gi Lee, Seung Hyuk Yim, Daham Kim, Hyunji Kim, Seonhyang Jeong, Sang Geun Jung, Young Suk Jo, Jandee Lee

**Affiliations:** 1 Department of Internal Medicine, Yonsei Cancer Center, Severance Hospital, Yonsei University College of Medicine, Seoul 120-752, Korea; lewoky7@yuhs.ac (W.K.L.); ljd0906@naver.com (D.K.); bambi_89@yuhs.ac (S.J.); 2 Brain Korea 21 PLUS Project for Medical Science, Yonsei University, Seoul 120-752, Korea; seulgi@yuhs.ac; 3 Department of Surgery, Yonsei Cancer Center, Severance Hospital, Yonsei University College of Medicine, Seoul 120-752, Korea; ljd0906@gmail.com (S.H.Y.); hjkim0612@yuhs.ac (H.K.); 4 Department of Gynecological Oncology, Bundang CHA Medical Center, CHA University, Seongnam, Gyeonggi-do 13496, Korea; jandee@yonsei.ac.kr

**Keywords:** hedgehog proteins, neoplasm invasiveness, thyroid cancer

## Abstract

Locally advanced thyroid cancer exhibits aggressive clinical features requiring extensive neck dissection. Therefore, it is important to identify changes in the tumor biology before local progression. Here, whole exome sequencing (WES) using tissues from locally advanced papillary thyroid cancer (PTC) presented a large number of single nucleotide variants (SNVs) in the metastatic lymph node (MLN), but not in normal tissues and primary tumors. Among those MLN-specific SNVs, a novel HHIP G516R (G1546A) mutation was also observed. Interestingly, in-depth analysis for exome sequencing data from the primary tumor presented altered nucleotide ‘A’ at a very low frequency indicating intra-tumor heterogeneity between the primary tumor and MLN. Computational prediction models such as PROVEAN and Polyphen suggested that HHIP G516R might affect protein function and stability. In vitro, HHIP G516R increased cell proliferation and promoted cell migration in thyroid cancer cells. HHIP G516R, a missense mutation, could be a representative example for the intra-tumor heterogeneity of locally advanced thyroid cancer, which can be a potential future therapeutic target for this disease.

## 1. Introduction

Thyroid cancer is the most common endocrine malignancy and is associated with an excellent long-term prognosis [1]. However, in a significant number of patients with papillary thyroid cancer (PTC), the disease displays a more aggressive behavior, including locoregional recurrence frequently associated with metastatic lymph node metastasis (LNM) [2,3]. In line with these observational data, prediction of LNM in patients with PTC has emerged as a research field in thyroid cancer. Moreover, in addition to clinicopathological poor prognostic parameters, molecular markers easily applied to fine-needle aspiration biopsy (FNAB) samples have been extensively investigated to determine the surgical extent, such as in prophylactic central lymph node dissection (CLND) [3,4,5]. Currently, the BRAF^V600E^ mutation is the most useful diagnostic and prognostic molecular marker in patients with PTC for predicting occult lymph node metastasis [5]. Actually, several meta-analyses recently reported an overall correlation of the BRAF^V600E^ mutation with extra-thyroidal extension (ETE) and LNM [6,7]. However, the results across individual studies are inconsistent, and several similar large retrospective trials have failed to corroborate these results [8,9]. Therefore, the overall significance of the BRAF^V600E^ mutation remains under debate.

Whole-exome sequencing (WES), a next-generation sequencing (NGS) technology, is an efficient approach to selectively identify cancer mutations in the coding regions of the genome, allowing the identification of potential causative alterations in unexpected genes. Although, compared to whole-genome sequencing (WGS), WES cannot identify most intronic and regulatory regions, it improves sequencing coverage and depth for coding regions, thus providing high sensitivity for uncovering even low-frequency mutations, as recently implemented by The Cancer Genome Atlas (TCGA) NIH project (http://cancergenome.nih.gov/). Thus, for basic research and molecular diagnostic applications, WES is currently the best NGS strategy considering its extensiveness, time required, and cost [10,11].

The Hedgehog (HH) signaling pathway was originally identified as a crucial pathway in embryonic patterning and development [12]. The central components of the mammalian HH pathway consist of three secretory ligands (Sonic Hedgehog (SHH), Indian Hedgehog (IHH), and Desert Hedgehog (DHH)); a 12-pass transmembrane receptor involved in the negative regulation of the pathway, Patched1 (PTCH1); a negative regulator, Hedgehog interacting protein (HHIP); a G-protein-coupled receptor like seven-pass transmembrane protein, Smoothened (SMO); and three transcription factors (GLI1, GLI2, and GLI3) [12]. Aberrant activation of the HH pathway has been linked to tumorigenesis in medulloblastoma [13] and basal cell carcinoma [14]. Moreover, hyperactive HH signaling has been identified in lymphoma, breast, prostate, colorectal, liver, stomach, and small cell lung cancers, as well as multiple myeloma and chronic myeloid leukemia [15,16]. Recently, the pathway was reported to be associated with thyroid cancer cell proliferation and invasiveness [17,18]. However, the role of HHIP in cancer is poorly understood, although it is well known that inactivating mutations in PTCH1 or Suppressor of fused homolog (SUFU) or activating mutations in SMO contribute to tumorigenesis through HH signaling activation [19,20].

Here, we performed WES of three locally advanced PTC cases, including normal tissue, the primary tumor, and metastatic lymph nodes, to identify additional driver mutations as well as mutations potentially facilitating tumor progression, such as LNM. We found a novel HHIP G516R mutation in the metastatic lymph node (MLN) and predicted the mutation-induced functional changes in HHIP, including protein function and physical properties, using several computational models for the functional effects of single nucleotide variants (SNVs). In addition, we observed the biological effects of this mutation on thyroid cancer cell properties in vitro.

## 2. Results

### 2.1. Spectrum and Characteristics of Somatic Mutations

**Figure 1:**
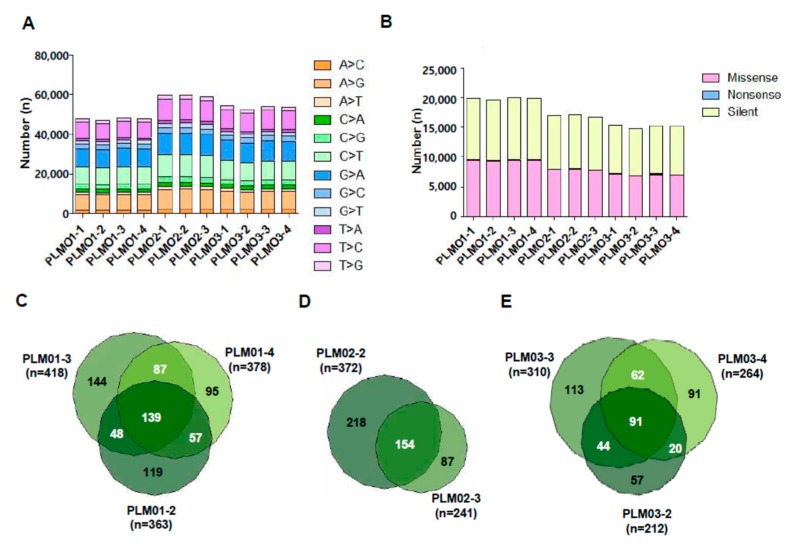
Total number of single nucleotide variants (SNVs) from whole exome sequencing of 11 tissues samples from three patients with papillary thyroid cancer (PTC). (**A**) Patterns of nucleotide substitution indicating the dominance of C > T/G > A/T > C/A > G transitions; (**B**) The numbers of missense, nonsense, and silent substitutions; (**C**–**E**) The numbers of common and sample-specific SNVs in PLM01 (**C**), PLM02 (**D**), and PLM03 (**E**).

The baseline characteristics of patients with PTC subjected to WES are summarized in Table S1. The total number of detected insertion, deletion, and single nucleotide variants (SNVs) and the numbers of common and sample-specific somatic insertions, deletions, and SNVs in primary tumors or metastatic lymph nodes are presented in Figure S1. The proper read rates ranged from 87.48 to 91.77%, and the properly paired rates ranged from 98.63% to 98.89%. A mean coverage depth of 108.7× per sample was achieved, with 89.2% of targets covered at a depth of ≥20×. In all samples, C > T/G > A/T > C/A > G transitions were dominant in the SNV substitution spectrum (70.46–71.24% of all mutations, Figure 1A), and the percentage of missense SNVs in all exon SNVs ranged from 46.22 to 47.31% (Figure 1B). Next, we calculated the number of somatic SNVs in primary tumors (PLM01-2, PLM02-2, and PLM03-2) and metastatic lymph nodes (PLM01-3 (level VI), PLM01-4 (level II), PLM02-3 (level IV), PLM03-3 (level VI), and PLM03-4 (level IV)) by removing common SNVs detected in each corresponding normal tissue (PLM01-1, PLM02-1, and PLM03-1). In this analysis, we found 139 common somatic SNVs among PLM01-2, PLM01-3, and PLM01-4 (Figure 1C) and 154 common somatic SNVs between PLM02-2 and PLM02-3 (Figure 1D). In case 3, we found 91 common somatic SNVs among PLM03-2, PLM03-3, and PLM03-4 (Figure 1E). The number of variants across the samples was independent of disease stage. Notably, we identified novel tumor- or lymph node-specific somatic mutations (Tables S2–S5 and Figure S2A).

### 2.2. A Novel Hedgehog Interacting protein (HHIP) G516R Mutation in Metastatic Lymph Nodes

**Figure 2:**
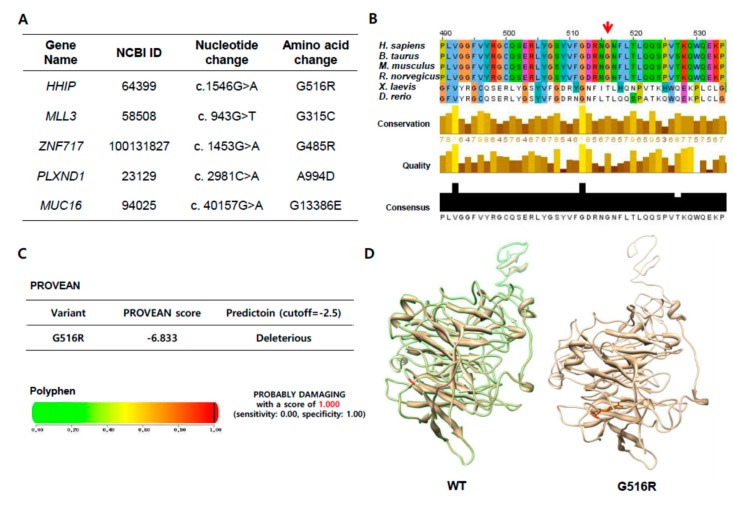
Analysis of novel tumor or metastatic lymph node-specific SNVs. (**A**) Five representative SNVs; (**B**) Sequence alignment of HHIP mutation sites in various species. The arrow indicates the amino acid at the site where the mutation was found in this study; (**C**) Results of PROVEAN and PolyPhen analyses of HHIP G516R; (**D**) Comparison of computational modeling between HHIP WT and HHIP G516R. Red color also indicates the amino acid at the site where the mutation was found in this study.

Among the gene list of novel somatic mutations in tumors or lymph nodes, we focused on a novel G1546A mutation in HHIP because its Polymorphism Phenotyping v2 (PolyPhen-2, http://genetics.bwh.harvard.edu/pph2/) and Protein Variation Effect Analyzer (PROVEAN, http://provean.jcvi.org/index.php) scores were highest among newly detected point mutations (Figure 2A and Table S6). This missense mutation is in *HHIP* exon 9, resulting in the substitution of glycine by arginine at position 516 (G516R). To verify the presence of the *HHIP* G1546A mutation, we performed pyrosequencing representing the mutant nucleotide “A” significantly on pyrograms using genomic DNA samples derived from MLN (PLM01-3 and PLM01-4) but not from normal and primary tumor tissues showing non-specific “A” peak below 5 percent (PLM01-1 and PLM01-2) (Figure S2). Interestingly, even though our WES analysis and pyrogram did not show the *HHIP* G1546A mutation in PLM01-2, an in-depth analysis indicated the mutant nucleotide “A,” suggesting that a small proportion of tumor cells might harbor the *HHIP* G1546A mutation (Figure S3) and indicating intra-tumor heterogeneity among genetically defined subclones. The 516 site was conserved among all vertebrate HHIP sequences queried (Figure 2B), but not in Xenopus and Drosophile, substantiating it as an evolutionary acquisition residue (Clustalw2, http://www.ebi.ac.uk/Tools/msa/clustalw2/). As listed in Table S6, PROVEAN predicted that G516R substitution affects protein function based on sequence homology and the physical properties of amino acids. Remarkably, the PROVEAN score was −6.833, definitely below the predefined threshold (−2.5), predicting a “deleterious effect” of the G516R mutation (Figure 2C). Moreover, PolyPhen-2 also indicated that the G516R mutation has probably been damaged, with a score of 1.000 (Specificity = 1.00, Figure 2C). I-MUTANT2.0 and I-MUTANT3.0 (http://folding.biofold.org/i-mutant/) suggested that the G516R mutation might affect protein stability and be a disease-related mutation (DDG value = −0.85, Table S6). Computational analysis of the HHIP extracellular domain (ECD) sequence revealed four globular domains: a cysteine-rich N-terminal domain with a Frizzled (Fz) fold, a central six-bladed β-propeller, and two C-terminal EGF repeats. The 516 residue is on the β-propeller domain, and the substitution of glycine by arginine changes the hydropathy index from −0.4 to −4.5 and allows the possession of a long side chain (Figure 2D). In summary, the HHIP G516R mutant was predicted to severely affect the protein structure and function.

### 2.3. HHIP Expression as a Marker of Hedgehog Signal Activation in Human Thyroid Cancer

**Figure 3:**
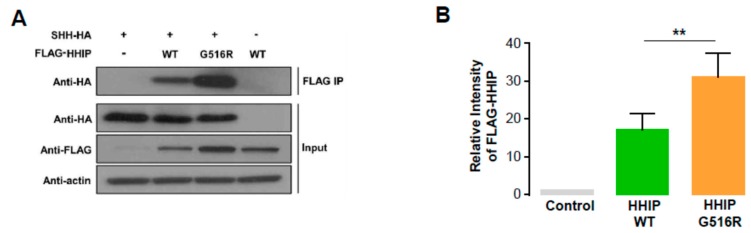
Protein to protein interaction of HHIP WT and G516R with SHH in human thyroid cancer. (**A**) Co-immunoprecipitation assay to investigate the interaction between SHH and HHIP WT or G516R mutant; (**B**) The quantification of FLAG-HHIP amount from triplicate experiments. Significant difference was decided by Mann–Whitney *U* test and is noted by asterisks (** *p* < 0.01). Data represent the mean ± SD.

Because HHIP is a well-known binding partner of SHH and the HHIP G516R mutant was predicted to severely affect the protein structure and function, we decided to perform co-immunoprecipitation assay using SHH-HA, FLAG-HHIP-WT, and FLAG-HHIP-G516R. First we transfected 8505C cells with indicated plasmids for 24 h and conducted co-immunoprecipitation with anti-FLAG antibody and detect an HA signal in precipitated pellets by western blot analysis (See Materials and Methods in detail) to see the impact of HHIP-G516R mutation on the interaction with SHH-HA. Interestingly, SHH-HA band intensity was not decreased in HHIP G516R transfected pellets compared to HHIP WT transfected pellets, indicating the HHIP-G516R mutant did not make any disturbance of the interaction with SHH compared to HHIP-WT (Figure 3). The other interesting finding was that the FLAG signal was much higher in HHIP G516R transfected lysates compared to HHIP-WT transfected lysates (Figure 3 and Figure S4), indicating interaction of the G516R mutant protein with SHH might increase the HHIP protein amount in thyroid cancer cells.

### 2.4. HHIP G516R Mutation Promotes Thyroid Cancer Cell Proliferation and Migration

**Figure 4:**
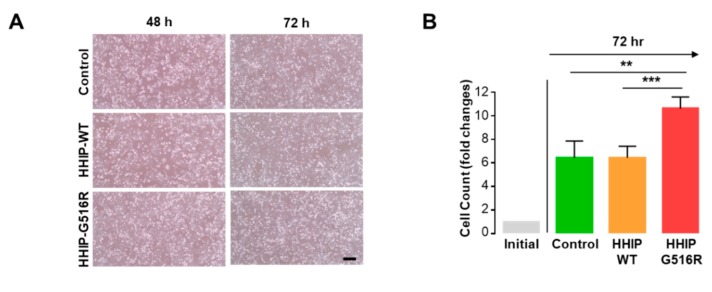

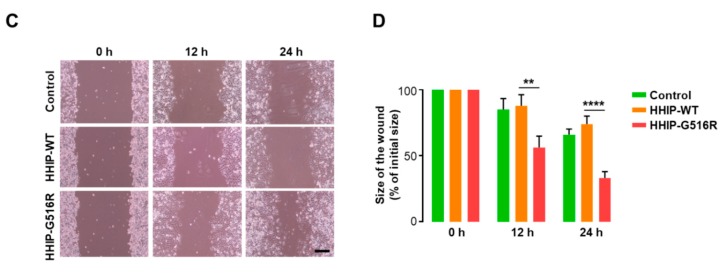
Contribution of the HHIP G516R mutation to thyroid cancer cell proliferation and migration. (**A**,**B**) Analysis of cell proliferation in 8505C cells transfected with HHIP WT or the G516R mutant. Representative microscopic images (**A**) and cell counting (**B**) for each condition (*n* = 4); (**C**,**D**) Live wound healing/migration assay in 8505C cells transfected with HHIP WT or the G516R mutant. Representative microscopic images (**C**) and quantitative analyses (**D**) for each condition (*n* = 4). Scale bar represents 200 μm. Significant differences were decided by two-tailed Mann–Whitney *U* test and are noted by asterisks (** *p* < 0.01, *** *p* < 0.001, and **** *p* < 0.0001). Data represent the mean ± SD.

Although our computational modeling indicated HHIP G516R mutation can affect the protein structure and function, we could not find any disturbance of interaction between HHIP G516R with SHH disappointingly. Next, we determined to investigate the functional effect of the HHIP G516R mutation on thyroid cancer cell properties to understand the biological role of this mutation in human thyroid cancer. To achieve this goal, we transfected 8505C cells with FLAG-HHIP WT and HHIP G516R mutant plasmid as indicated (Figure 4 and Figure S5). To observe the effect of the HHIP G516R mutation on proliferation, 8505C cells were analyzed by cell counting 72 h after the respective transfection. As expected, the HHIP G516R mutation significantly promoted cell proliferation even though the overexpression of HHIP-WT did not have any effect (Figure 4A,B and Figure S5). We next performed a wound-healing/migration assay to investigate the effect of the HHIP G516R mutation on thyroid cancer cell behavior. Interestingly, 12 or 24 h after the monolayers were scratched, the sizes of the wounds were significantly smaller in 8505C cells transfected with HHIP-G516R than in cells transfected with the control vector or HHIP-WT (Figure 4C,D and Figure S5), indicating that the HHIP G516R mutation promotes thyroid cancer cell migration. All these data indicate that the HHIP G516R mutation promotes tumor aggressiveness in thyroid cancer compared to HHIP-WT.

## 3. Discussion

In this study, we identified the novel HHIP G516R mutation by performing WES of locally advanced thyroid cancer tissues, including primary tumors and metastatic lymph nodes. This mutation was predicted to have severe detrimental effects on HHIP function. In fact, in vitro experiments showed that HHIP G516R promoted thyroid cancer cell proliferation and migration.

Of note, our pyrosequencing and in-depth analysis suggested that acquisition of intra-tumor heterogeneity might be a crucial process for tumor progression. Cancers contain numerous clones with various population sizes, different genetic development, and distinct phenotypic features [21,22]. This intra-tumor heterogeneity drives carcinogenesis and tumor progression and contribute to therapeutic failure and acquired drug resistance [21]. WES, which is currently one of the best NGS strategies considering its cost-effectiveness, is a powerful analysis for unraveling the clonal heterogeneity, evolution, and potential for competitive generation of resistant subclones [23]. Here, the proportion of mutant nucleotide A (HHIP G516R mutation) was much higher in metastatic lymph nodes than in primary tumors (Figure S2B–D, 3.5% versus 15.4% or 25.8%). In fact, 3.5% is below the positive cut-off value for pyrosequencing. Furthermore, the in-depth analysis demonstrated that the mutant nucleotide A existed in primary tumors although WES analysis could not identify the HHIP G1546A mutation (Figure S3). These findings suggest that a small population of tumor cells harboring a specific mutation like HHIP G1546A could evolve during the invasion and metastatic process, as indicated by the increased proportion of mutant nucleotide A.

Aberrant activation of HH signaling is closely related to several types of cancer, although this pathway is crucial for many steps in embryonic development [19]. Similar to PTCH1, HHIP is a negative regulator of the HH pathway and is equipotent against three mammalian HH ligands, SHH, IHH, and DHH [24]. According to a recent study, HHIP competitively binds at the SHH pseudo-active site against PTCH1, thereby inhibiting the HH pathway [25]. Our computational modeling suggested that HHIP G516R might have significant structural changes induced by the single amino acid substitution, being predicted to affect protein-to-protein interactions. However, the interaction of HHIP with SHH was not affected by G516R substitution in our co-immunoprecipitation (co-IP) assay. We understand that this negative co-IP data indicated that a non-canonical pathway might be operational in PTC harboring HHIP G516R because this novel mutation was able to increase cell proliferation and migration even though HHIP WT has no effect on these tumor cell phenotypes. In general, decreased HHIP expression has been indicated in several tumors, suggesting a potential role of HHIP in tumor suppression [26]. Several recent studies reported that HHIP is hypermethylated and transcriptionally down-regulated in some cancers and mouse models [27,28]. However, our western blot data also indicated that the amount of HHIP G516R protein was not decreased, compared to HHIP WT protein. This finding could also be evidence supporting the existence of a non-canonical pathway operated by mutant HHIP G516R.

Our study has some limitations. Firstly, because of the small number of tumor samples, the findings from this study need to be validated in future studies. However, our study might be a representative model to suggest the presence of intra-tumor heterogeneity in locally advanced thyroid cancers. In addition, the functional study of mutant HHIP G516R in this paper was only performed by transient transfection technology. Although the direct effect of mutant protein can be best investigated using this technique, the long-term effects of mutant HHIP G516R also need to be validated. Despite this, the mutation was detected in human locally advanced thyroid cancers, which might suggest the long-term effect of mutant HHIP G516R. Thirdly, although our data indicated the existence of intra-tumor heterogeneity, novel technology platforms such as the study of genomics, transcriptomics, proteomics, and metabolomics at the single cell level will shed light on the poorly understood areas in this field.

In conclusion, WES indicated intra-tumor heterogeneity in locally advanced thyroid cancers. In addition, this study first identified HHIP G516R (G1546A), which promotes tumor aggressiveness in thyroid cancer cells.

## 4. Materials and Methods

### 4.1. Whole Exome Sequencing

Tissue samples from three patients with locally advanced PTC were obtained by surgery for DNA extraction in April 2014. The tissues were collected in cryogenic tubes and were stored at −20°C in DNAlater solution (Thermo Fisher Scientific, Inc., Waltham, MA, USA). DNA extraction was performed using the QiaAmp DNA Mini kit (Qiagen, Inc., Valencia, CA, USA). Eleven samples were analyzed by WES, performed at 200 × 2 bp on a HiScanSQ Illumina platform (Illumina, Inc., San Diego, CA, USA). The cleaned reads were mapped to the human reference genome hg38 using Burrows–Whealer Aligner in paired-end mode [29]. To remove the optical and PCR duplicates and optimize the alignment around InDels, Samtools and GATK were used [30,31]. Single nucleotide variants (SNVs) and InDels were detected using GATK (HaplotypeCaller mode), Mutect, and SNVmix2 [32,33]. The whole set of detected variants was refined filtering threshold for each sample, based on the presence and relative enrichment of the BRAF^V600E^ mutation; only samples with >10% tumor vs. normal allele were included in the study. The obtained variants were annotated with 1000 Genomes allele frequencies, dbSNP 149 rsIDs, Exac, and EVS using Annovar and Oncotator [34,35]. Finally, to predict the pathogenic significance of the emerging variants, mutation-prediction tools (PolyPhen2, Proven and SIFT) were used [36,37,38]. Patient information and clinicopathological parameters were analyzed retrospectively. Written informed consent was obtained from the patients and all protocols were approved by the institutional review board of Severance Hospital (The approval number, 4-2013-0546; The approval date, 7 April 2013).

### 4.2. Pyrosequencing

Pyrosequencing to verify the HHIP G516R mutation in tissue samples was performed as previously described [39]. Briefly, A 5-µL volume of the genomic DNA was amplified by PCR using standard conditions (95 °C: 5 min; 94 °C: 30 s, 68 °C: 30 s, 72 °C: 30 s, for 32 cycles; 70 °C: 10 min) with the following primers: forward 5′-GAA GCT ACG TGT TTG GAG ATC G-3′ and reverse 5′ biotin-GCA GTT TGC CAA ATG ATT AAT G-3′. After immobilizing the resulting PCR products onto magnetic streptavidin-coated beads (Magnetic Biosolutions, Stockholm, Sweden), the bead/DNA complex was washed to remove unwanted components. A pyrosequencing primer (5′-CGT GTT TGG AGA TCG TA-3′) was added and annealed to the captured strand following the manufacturer’s instructions (Magnetic Biosolutions) with Magnatrix 1200 robot (Magnetic Biosolutions). The primed single-stranded DNA templates were transferred to a PSQ HS 96 A Pyrosequencer (Biotage, Uppsala, Sweden).

### 4.3. Computational Analysis of the HHIP Extracellular Domain

The protein sequence of human HHIP (NP_071920.1) was obtained from NCBI Human Genome Resources (http://www.ncbi.nlm.nih.gov/protein). Computational models were generated using MODELLER [40], and a reliable structure was selected by PROCHECK analysis [41]. The predicted model quality was checked assessed by Ramachandran plot analysis [42].

### 4.4. Cell Culture

The 8505C human thyroid cancer cell line was kindly provided by Peter A Kopp (Northwestern University, Chicago, IL, USA). 8505C cells were cultured in RPMI-1640 (Corning, VA, USA) media containing 10% fetal bovine serum and 1% penicillin/streptomycin in a humidified atmosphere of 5% CO_2_ and 95% air at 37 °C.

### 4.5. Transfection of HHIP Wild-Type and G516R Mutant Plasmids

pcDNA3.1-FLAG-HHIP wild-type (WT) was purchased from Origene (Rockville, MD, USA), and pcDNA3.1-FLAG-HHIP-G516R mutant plasmid was generated by Site-Directed Mutagenesis Kits according to the manufacturer’s protocol (Thermo Fisher Scientific, Waltham, MA, USA). To determine the effects of the HHIP G516R mutation on cancer cell properties, 8505C cells were transfected with pcDNA3.1 control vector, pcDNA3.1-FLAG-HHIP WT, or pcDNA3.1-FLAG-HHIP G516R mutant using Lipofectamine 3000 reagent (Invitrogen, Carlsbad, CA, USA). The cells were analyzed by western blotting, cell counting, and wound healing/migration assay 48 or 72 h after the transfection.

### 4.6. Western Blot Analysis

Cells were washed once with ice-cold PBS and immediately lysed with cell extraction buffer (FNN0011, Invitrogen) and EDTA-free protease inhibitor (1 tablet per 25 mL buffer, Roche, Basel, Switzerland). The cell lysates were incubated at 4 °C for 30 min and cleared by centrifugation at 13,000 rpm at 4 °C for 10 min. Then, 20–30 μg of proteins from the cell lysates was prepared, resolved on 8% SDS-PAGE gels, and transferred to PVDF membranes. The membranes were probed with antibodies against FLAG (A8592, Sigma, St. Louis, MO, USA), HA (3724S, Cell Signaling, Danvers, MA, USA), and β-actin (sc-47778, Santa Cruz Biotechnology, Inc., Dallas, TX, USA).

### 4.7. Co-Immunoprecipitation Assay

For co-immunoprecipitation (IP) assays to evaluate the interaction between FLAG-HHIP and SHH-HA, we first transfected 8505C cells with SHH-HA and/or FLAG-HHIP-WT, or FLAG-HHIP-G516R plasmid as indicated. After 24 h incubation at normal culture condition, the cell extracts from the transfected cells were cleared by centrifugation at 13,000 rpm at 4 °C for 10 min, and the supernatants were incubated with anti-FLAG antibody (F1804, Sigma) for 12 h at 4 °C under gentle agitation. In order to isolate the protein complex binding with anti-FLAG antibody, protein A/G PLUS-Agarose (sc-2003, Santa Cruz) was then added to each sample, and the mixtures were incubated overnight at 4 °C on a rotating device. Immunoprecipitates formed by this process were collected by centrifugation at 1000× *g* for 5 min at 4 °C and washed three times with PBS. The pellets were eluted by heating at 95 °C for 5 min in 1× electrophoresis sample buffer for western blot analysis as indicated.

### 4.8. Wound Healing/Migration Assay

8505C cells were seeded in 6 well culture plates and allowed to form a confluent monolayer. A scratch wound was made with a 1000 μL pipette tip. The cells were then washed with PBS to remove cell debris. Microscopic images were obtained 0, 12, and 24 h after scratching. The sizes of the wounds were measured using Image J software (https://imagej.nih.gov/ij/). The data are shown as a percentage of the initial size. The assay was performed in quadruplicate in each condition.

### 4.9. Statistical Analysis

Continuous variables were compared using two-tailed Student’s *t* test, and categorical data were compared using the *x*^2^ test or Fisher’s exact test. Statistical analyses were performed using SPSS software for Windows (Version 23; IBM Corp., New York, NY, USA) or GraphPad Prism 6 (GraphPad Software Inc., San Diego, CA, USA). *p* values less than 0.05 were considered statistically significant.

## Supplementary Materials

Supplementary materials can be found at http://www.mdpi.com/1422-0067/19/10/2867/s1.
