# Supplementary material for: Whole Exome Sequencing Identifies a Novel Hedgehog-Interacting Protein G516R Mutation in Locally Advanced Papillary Thyroid Cancer
Source: Int J Mol Sci. 2018 Sep 21;19(10):2867. doi: 10.3390/ijms19102867 (PMC6213497; doi:10.3390/ijms19102867)
Supplement: Supplementary file 1 [file ijms-19-02867-s001.zip › ijms-347887-for final check-Supplementary Material.pdf]

Supplementary Materials

This file includes:

Figures S1–5;

Tables S1, 2 and 6;

Tables S3–5 see excel files.

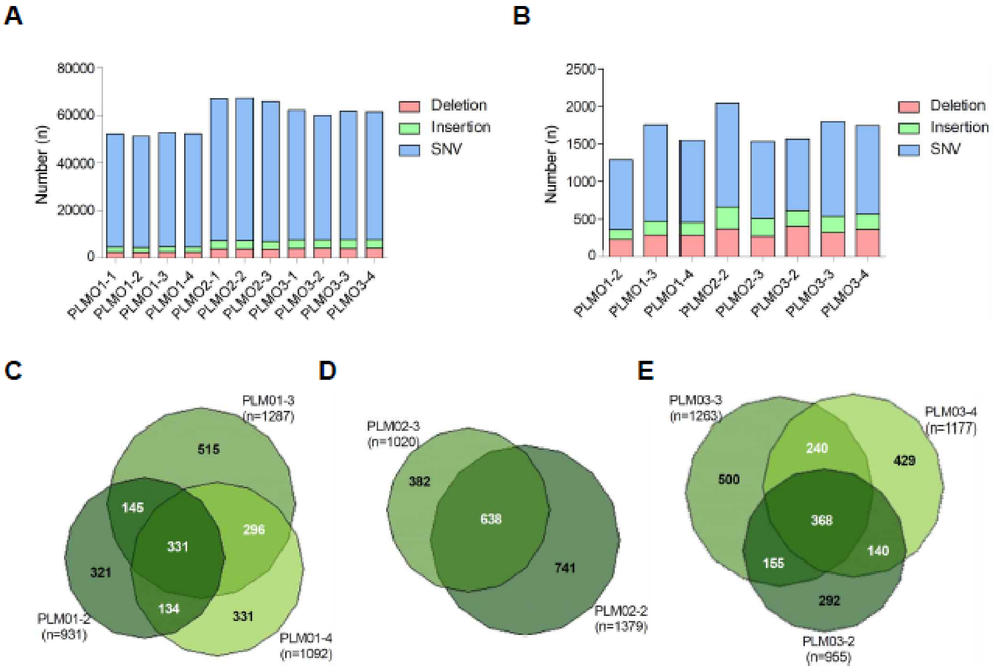

Figure S1. Spectrum and characteristics of whole exome sequencing in locally advanced thyroid cancers. **(A)** The total numbers of deletions, insertions, and SNVs detected in 11 tissue samples from three patients. (**B)** The numbers of common and sample-specific deletions, insertions, and SNVs in primary tumors or metastatic lymph nodes. (**C–E**) The numbers of common and sample-specific deletions, insertions, and SNVs in PLM01 (**C**), PLM02 (**D**), and PLM03 (**E**).

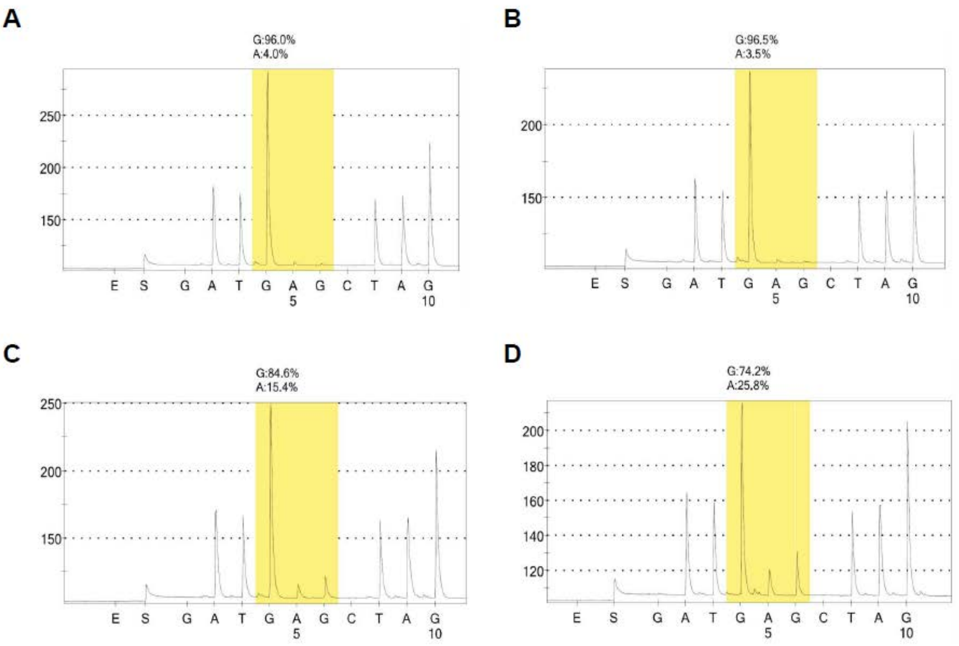

Figure S2. Pyrograms to detect the HHIP G516R mutation from PLM01-1 (A), PLM01-2 (B), PLM01-3 (C), and PLM01-4 (D). The percentages of each nucleotide (G or A) are indicated in the heading of each pyrogram.

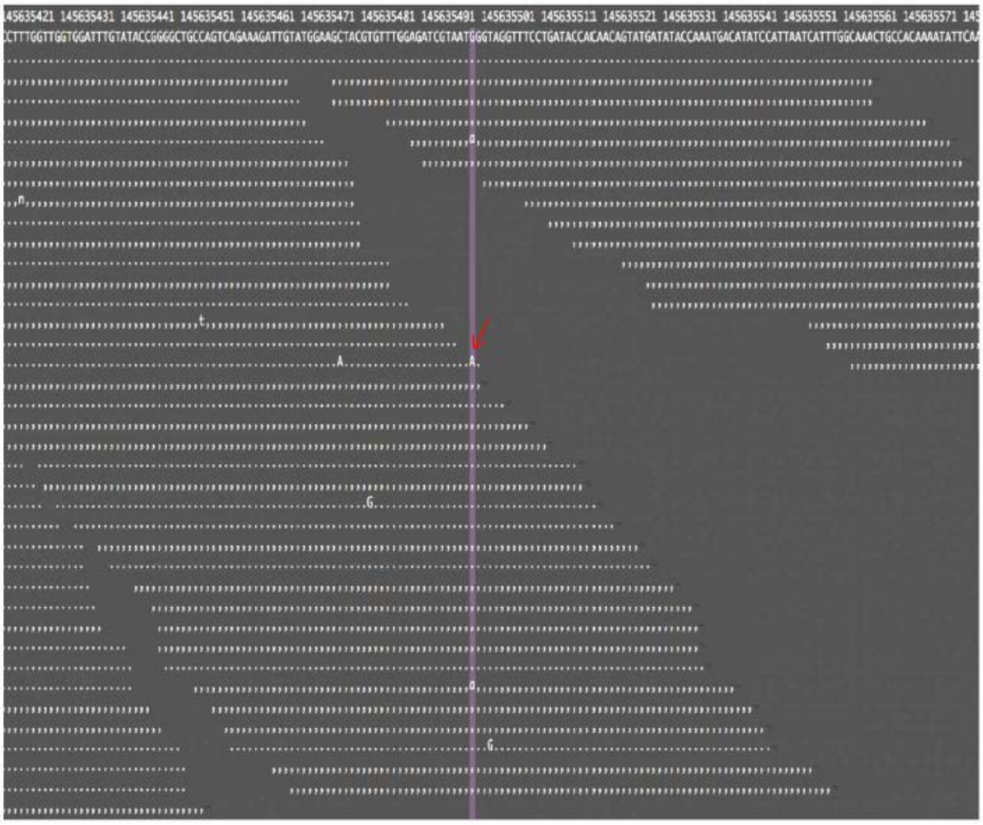

Figure S3. Sequence alignment results indicating the presence of a mutant allele below the variant calling threshold in PLM01-2. The arrow indicates the mutant allele “A.”

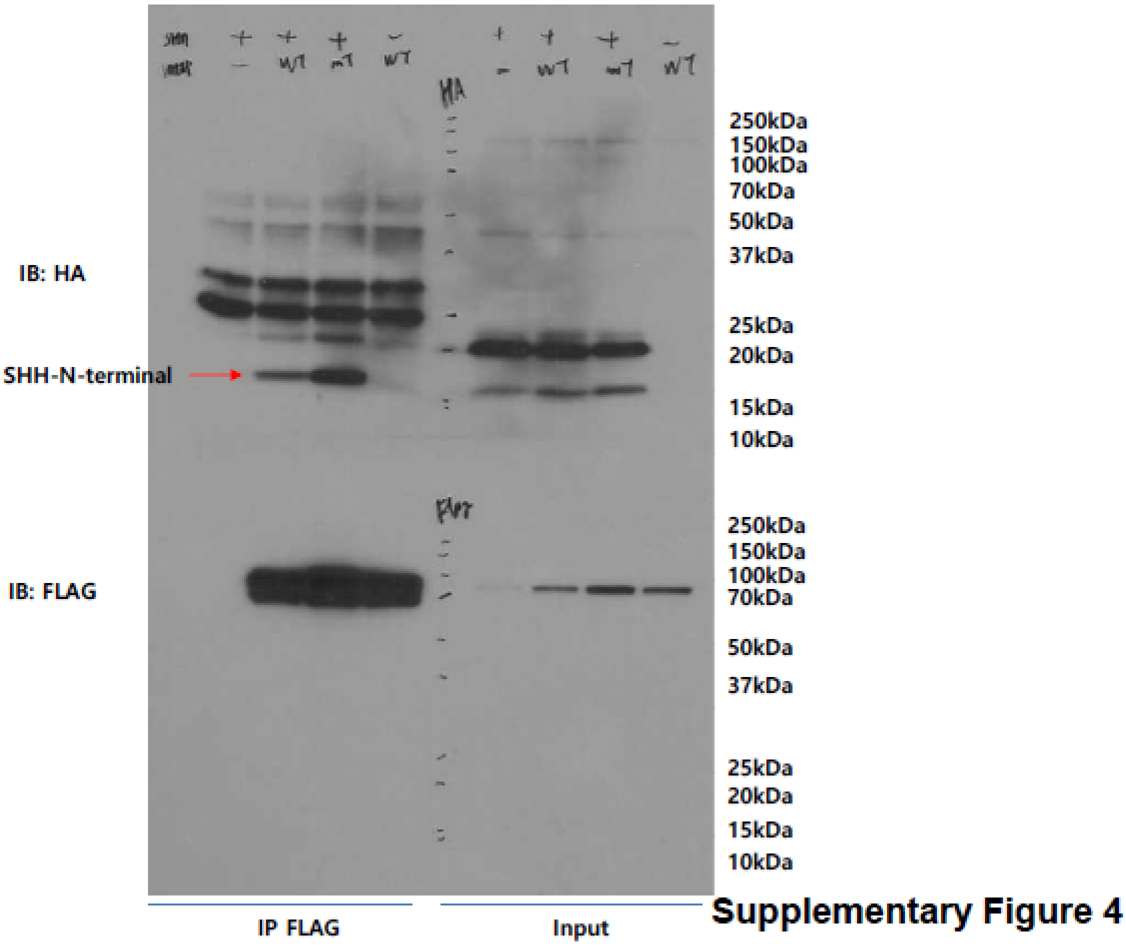

Figure S4. Western blot analysis to show the amount of FLAG-HHIP and SHH-HA protein immuno-precipitated (IP) by anti-FLAG antibody and to show the expression of FLAG-HHIP and SHH-HA protein in the cells transfected as indicated.

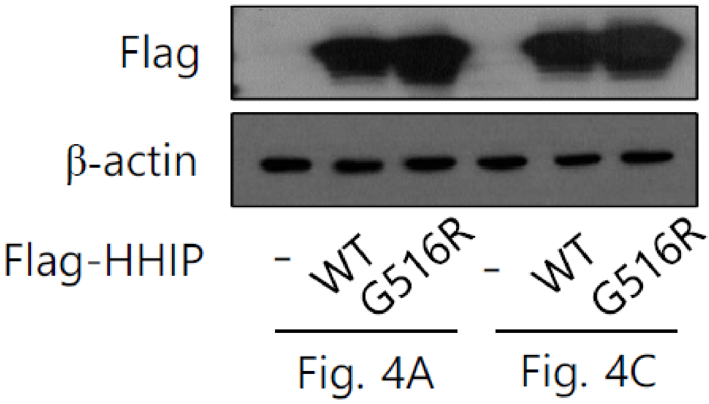

Figure S5. Western blot analysis to show FLAG-HHIP expression in the cells transfected with FLAG-HHIP-WT or FLAG-HHIP-G516R.

Table S1. Clinicopathological characteristics of patients with papillary thyroid cancer subjected to whole exome sequencing.

Case Number	1 (PLM01)	2 (PLM02)	3 (PLM03)
Age (yr)	33	31	30
Tumor size (cm)	1.92	4.0	3.0
T-stage	T4	T3	T4
Extrathyroidal extension	moderate	minimal	moderate
Multifocality	multifocal	unifocal	unifocal
Metastatic lymph node (MLN)	N1b	N1b	N1b
Distant metastasis	M0	M0	M0
TNM stage group	I	I	I
Tissue labeling	PLM01-1; Normal<br>PLM01-2; Tumor<br>PLM01-3;<br>Level VI MLN<br>PLM01-4;<br>Level II MLN	PLM02-1; Normal<br>PLM02-2; Tumor<br>PLM02-3;<br>Level IV MLN	PLM03-1; Normal<br>PLM03-2; Tumor<br>PLM03-3;<br>Level VI MLN<br>PLM03-4;<br>Level IV MLN

Table S2. Gene list of novel somatic SNVs in tumor tissues or metastatic lymph nodes.

Name	NCBI ID	Description	Nucleotide change	Amino acid change
AHNAK2	113146	AHNAK nucleoprotein 2	c. 3211C>G	p. Lue1071Val
AHNAK2	113146	AHNAK nucleoprotein 2	c. 3553T>A	p. Ser1185Thr
AHNAK2	113146	AHNAK nucleoprotein 2	c. 3669C>G	p. His1223Gln
ANKRD36	375248	ankyrin repeat domain 36	c. 844G>A	p. Glu282Lys
ANKRD36	375248	ankyrin repeat domain 36	c. 853A>G	p. Ile285Val
ANKRD40	91369	ankyrin repeat domain 40	c. 294A>T	p. Glu98Asp
AQP7	364	aquaporin 7	c. 650T>A	p. Leu217His
AQP7	364	aquaporin 7	c. 657G>A	p. Met219Ile
AQP7	364	aquaporin 7	c. 797A>C	p. Tyr266Ser
ASPG	374569	asparaginase	c. 76C>T	p. Leu26Phe
BAGE2	85319	B melanoma antigen family, member 2B melanoma antigen family, member 2	c. 5C>G	p. Ala2Gly
CCKAR	886	cholecystokinin A receptor	c. 1220G>A	p. Gly407Glu
CDC27	996	cell division cycle 27	c. 259G>C	p. Glu87Gln
CDC27	996	cell division cycle 27	c. 1400G>A	p. Gly467Asp
COPB2	9276	coatomer protein complex, subunit beta 2 (beta prime)	c. 193T>C	p. Phe65Leu
COPS7A	50813	COP9 signalosome subunit 7A	c. 790C>A	p. Leu264Ile
COPZ2	51226	coatomer protein complex, subunit zeta 2	c. 65G>C	p. Gly22Ala
CTBP2	1488	C-terminal binding protein 2	c. 593G>C	p. Arg198Pro
CTBP2	1488	C-terminal binding protein 2	c. 599A>C	p. Lys200Thr
DCP1B	196513	decapping mRNA 1B	c. 753C>G	p. His251Gln
DMKN	93099	dermokine	c. 846T>A	p. Ser 282Arg
DSC3	1825	desmocollin 3	c. 37G>T	p. Ala13Ser
FAM163A	148753	family with sequence similarity 163, member A	c. 275C>G	p. Ala92Gly
FAM186A	121006	family with sequence similarity 186, member A	c. 121006C>T	p. Pro1590Ser
FAM75C1	441452	SPATA31C1, SPATA31 subfamily C, member 1	c. 1958A>G	p. Glu653Gly
FLG	2312	filaggrin	c. 6697C>T	p. Pro2233Ser
GIGYF2	26058	GRB10 interacting GYF protein 2	c. 3650C>A	p. Pro1217Gln
GLG1	2734	golgi glycoprotein 1	c. 440C>T	p. Pro147Leu
GXYLT1	283464	glucoside xylosyltransferase 1	c. 224A>G	p. Tyr75Cys
GXYLT1	283464	glucoside xylosyltransferase 1	c. 228T>A	p. Ser76Arg
GXYLT1	283464	glucoside xylosyltransferase 1	c. 257A>T	p. His86Leu
HGC6.3	100128124	uncharacterized LOC100128124	c. 379G>A	p. Val127Ile
HGC6.3	100128124	uncharacterized LOC100128124	c. 493A>G	p. Thr165Ala
HHIP	64399	hedgehog interacting protein	c. 1546G>A	p. Gly516Arg
HLA-A	3105	major histocompatibility complex, class I, A	c. 296G>T	p. Arg99Leu
HLA-DRB1	3123	major histocompatibility complex, class II, DR beta 1	c. 97C>G	p. Arg33Gly
HLA-G	3135	major histocompatibility complex, class I,	c. 895A>G	p. Lys299Glu

		G		
IGSF3	3321	immunoglobulin superfamily, member 3	c. 1301A>G	p. Glu434Gly
IGSF3	3321	immunoglobulin superfamily, member 3	c. 1338G>C	p. Glu446Asp
IGSF3	3321	immunoglobulin superfamily, member 3	c.1465G>A	p. Val489Met
ILF3	3609	interleukin enhancer binding factor 3, 90kDa	c. 1789G>C	p. Asp597His
KCNN3	3782	potassium intermediate/small conductance calcium-activated channel, subfamily N, member 3	c. 197T>A	p. Leu66His
KRT6A	3853	keratin 6A	c. 745T>C	p. Phe249Leu
KRTAP4-11	653240	keratin associated protein 4-11	c. 277A>G	p. Met93Val
KRTAP4-8	728224	keratin associated protein 4-8	c. 204C>A	p. Ser68Arg
MAGEC1	9947	melanoma antigen family C, 1	c. 427A>C	p. Ile143Leu
MAML3	55534	mastermind-like 3 (Drosophila)	c. 1453A>C	p. Lys485Gln
MAP1LC3B	81631	microtubule-associated protein 1 light chain 3 beta	c. 338A>G	p. Tyr113Cys
MLL3	58508	KMT2C, lysine (K)-specific methyltransferase 2C	c. 943G>T	p. Gly315Cys
MUC16	94025	mucin 16, cell surface associated	c. 40157G>A	p. Gly13386Glu
MUC5B	727897	mucin 5B, oligomeric mucus/gel-form	c. 3290G>A	p. Arg1097His
MUC6	4588	mucin 6, oligomeric mucus/gel-forming	c. 4609G>A	p. Val1537Ile
MUC6	4588	mucin 6, oligomeric mucus/gel-forming	c. 4615C>A	p. Pro1539Thr
MUC6	4588	mucin 6, oligomeric mucus/gel-forming	c. 4706C>T	p. Pro1569Leu
MUC6	4588	mucin 6, oligomeric mucus/gel-forming	c. 5017C>G	p. Leu1673Val
MUC6	4588	mucin 6, oligomeric mucus/gel-forming	c. 5210G>C	p. Arg1737Pro
MUC6	4588	mucin 6, oligomeric mucus/gel-forming	c. 6218A>C	p. Gln2073Pro
MUC6	4588	mucin 6, oligomeric mucus/gel-forming	c. 6242C>G	p. Ala2081Gly
NCOR1	9611	nuclear receptor corepressor 1	c. 76C>A	p. Gln26Lys
OR8U8	504189	olfactory receptor, family 8, subfamily U, member 8	c. 162T>A	p. Ser54Arg
PABPC1	26986	poly(A) binding protein, cytoplasmic 1	c. 376C>G	p. Leu126Val
PCMTD1	115294	protein-L-isoaspartate (D-aspartate) O-methyltransferase domain containing 1	c. 1004G<C	p. Arg335Thr
PCMTD1	115294	protein-L-isoaspartate (D-aspartate) O-methyltransferase domain containing 1	c. 1024C<A	p. Pro342Thr
PDSS1	23590	prenyl (decaprenyl) diphosphate synthase, subunit 1	c. 83G>T	p. Arg28Leu
PHGR1	644844	proline/histidine/glycine-rich 1	c. 142G>C	p. Gly48Arg
PHGR1	644844	proline/histidine/glycine-rich 1	c. 143G>A	p. Gly48Asp
PLIN4	729359	perilipin 4	c. 270G>A	p. Val903Met
PLXND1	23129	plexin D1	c. 2981C>A	p. Ala994Asp
POLR3B	55703	polymerase (RNA) III (DNA directed) polypeptide B	c. 1102C>T	p. Leu368Phe
POTEC	388468	POTE ankyrin domain family, member C	c. 355G>A	p. Ala119Thr
PRDM9	56979	PRDM9 PR domain containing 9	c. 2524C>A	p. Arg842Ser
PRSS3	5646	protease, serine, 3	c. 685A>G	p. Lys229Glu
PRSS3	5646	protease, serine, 3	c. 835T>C	p. Trp279Arg
PRSS3	5646	protease, serine, 3	c. 836G>A	p. Trp279Stop

RBFOX3	146713	RNA binding protein, fox-1 homolog (C. elegans) 3	c. 22G>C	p. Ala8Pro
RP1L1	94137	retinitis pigmentosa 1-like 1	c. 4130T>C	p. Val1377Ala
SEZ6	124925	seizure related 6 homolog (mouse)	c. 2888G>C	p. Arg963Pro
SLC9B1	150159	solute carrier family 9, subfamily B (NHA1, cation proton antiporter 1), member 1	c. 837A>G	p. Ile279Met
SLC9B1	150159	solute carrier family 9, subfamily B (NHA1, cation proton antiporter 1), member 1	c. 859A>G	p. Ile287Val
SPATC1	375686	spermatogenesis and centriole associated 1	c. 979A>C	p. Thr327Pro
SPIRE2	84501	spire-type actin nucleation factor 2	c. 40G>A	p. Ala14Thr
SSC5D	284297	scavenger receptor cysteine rich domain containing (5 domains)	c. 3893C>T	p. His1300Tyr
SSC5D	284297	scavenger receptor cysteine rich domain containing (5 domains)	c. 3909G>A	p. Met1303Ile
SSC5D	284297	scavenger receptor cysteine rich domain containing (5 domains)	c. 3934T>G	p. Tyr1312Asp
TAS2R30	259293	taste receptor, type 2, member 30	c. 680C>T	p. Ala227Val
TAS2R30	259293	taste receptor, type 2, member 30	c. 727C>G	p. Leu243Val
TCHH	7062	trichohyalin	c. 1477G>C	p. Glu493Gln
TMEM200B	399474	transmembrane protein 200B	c.251C>T	p. Ser84Phe
TTLL4	9654	tubulin tyrosine ligase-like family, member 4	c. 1399A>C	p. Thr467Pro
TUBA3D	113457	tubulin, alpha 3d	c. 1040G>T	p. Cys347Phe
UBXN11	91544	UBX domain protein 11	c.1435T>G	p. Cys479Gly
USP6	9098	ubiquitin specific peptidase 6, (Tre-2 oncogene)	c. 201T>G	p. Ile67Met
USP6	9098	ubiquitin specific peptidase 6, (Tre-2 oncogene)	c. 202C>T	p. Arg68Trp
ZNF208	7757	zinc finger protein 208	c. 973G>T	p. Val325Phe
ZNF676	163223	zinc finger protein 676	c. 782G>C	p. Gly261Ala
ZNF717	100131827	zinc finger protein 717	c. 277G>A	p. Ala93Thr
ZNF717	100131827	zinc finger protein 717	c. 557C>T	p. Thr186Ile
ZNF717	100131827	zinc finger protein 717	c. 1453G>A	p. Gly485Arg
ZNF717	100131827	zinc finger protein 717	c. 1472A>G	p. Lys491Pro
ZNF717	100131827	zinc finger protein 717	c. 1475C>T	p. Ser492Leu
ZNF717	100131827	zinc finger protein 717	c. 1549C>T	p. Arg517Cys
ZNF717	100131827	zinc finger protein 717	c.2738T>G	p. Phe913Cys
ZNF880	400713	zinc finger protein 880	c. 947A>G	p. Lys316Arg
ZNF880	400713	zinc finger protein 880	c. 956G>C	p. Ser319Thr
ZXDB	158586	zinc finger, X-linked, duplicated B	c. 364G>A	p. Glu122Lys

Table S6. Prediction of the effect of novel mutations on protein function and stability.

SNV	Protein Acession	Polyphen score	PROVEAN score	I-Mutant 2.0 DDG (Kcal/mol)	I-Mutant 3.0 DDG (Kcal/mol)
AHNAK2 L1071V	NP_612429	0.000	-0.408	-1.53	-1.23
AHNAK2 S1185T	NP_612429	0.891	-1.225	-0.49	-0.33
AHNAK2 H1223Q	NP_612429	0.000	1.483	-0.13	-0.20
ANKRD36 E282K	NP_001157787	0.000	-0.629	-1.47	-0.56
ANKRD36 I285V	NP_001157787	0.000	-0.166	-1.54	-1.04
ANKRD40 E98D	NP_443087	0.253	-0.079	-0.90	-0.62
AQP7 Y266S	NP_001161	0.000	-1.502	-2.02	-1.37
AQP7 M219I	NP_001161	0.000	-1.527	-1.53	-0.87
AQP7 L217H	NP_001161	0.004	-3.814	-1.92	-2.06
ASPG L26F	NP_001073933	0.620	-0.533	-0.80	-1.06
BAGE2 A2G	NP_872288	NA	-3.400	-2.08	-1.32
CCKAR G407E	NP_000721	0.001	0.384	0.14	-0.23
CDC27 E87Q	NP_001107563	0.918	-2.126	-0.45	-0.53
CDC27 G467D	NP_001107563	0.999	-3.966	-1.39	-0.80
COPB2 F65L	NP_004757	0.999	-5.761	-0.63	-1.29
COPS7A L264I	NP_001157566	0.434	-0.744	-0.96	-0.68
COPZ2 G22A	NP_057513	0.119	-0.243	0.45	0.08
CTBP2 R198P	NP_001077383	0.729	-6.336	-1.06	-0.60
CTBP2 K200T	NP_001077383	0.994	-6.768	-0.80	-0.37
DCP1B H251Q	NP_689853	0.001	-1.144	1.26	0.29
DMKN S282R	NP_201574	0.571	-1.040	-0.39	-0.30
DSC3 A13S	NP_077741	0.008	-0.242	0.48	-0.53
FAM163A A92G	NP_775780	0.000	-0.358	-0.61	-0.93
FAM186A P1590S	NP_001138947	0.021	0.315	0.11	-0.71
FAM75C1 E653G	NP_001138596	NA	-3.388	-0.78	-0.91
FLG P2233S	NP_002007	0.001	1.911	-0.97	-1.32
GIGYF2 P1217Q	NP_056390	0.000	-0.177	1.01	0.00
GLG1 P147L	NP_036333	0.038	-0.937	-0.44	-0.61
GXYLT1 Y75C	NP_001093120	0.001	-1.851	1.19	-1.02
GXYLT1 S76R	NP_001093120	0.000	-0.643	-0.70	-0.05
GXYLT1 H86L	NP_001093120	0.937	-7.471	2.09	0.71
HGC6.3 V127I	NP_001123367	0.079	-0.547	-0.84	-0.34
HGC6.3 T165A	NP_001123367	0.005	0.328	-0.87	-0.79
HHIP G516R	NP_071920	1.000	-6.833	-0.53	-0.85
HLA-A R99L	NP_002107	0.002	-6.070	-0.46	-0.54
HLA-DRB1 R33G	NP_001230894	0.002	-2.452	-1.88	-1.61
HLA-G K299E	NP_002118	0.000	1.921	-0.33	-0.13
IGSF3 E434G	NP_001533	0.127	-1.529	-2.72	-1.36
IGSF3 E446D	NP_001533	0.000	-1.660	-0.23	-0.70
IGSF3 V489M	NP_001533	0.031	-1.494	-1.99	-0.99

ILF3 D597H	NP_036350	0.160	-0.350	-0.69	-0.60
KCNN3 L66H	NP_002240	0.004	-0.225	0.88	-0.21
KRT6A F249L	NP_005545	0.116	-4.186	-3.22	-1.46
KRTAP4-11 M93V	NP_149048	0.000	1.116	-0.13	-0.46
KRTAP4-8 S68R	NP_114166	0.004	-3.603	-0.37	-0.08
MAGEC1 I143L	NP_005453	0.001	0.019	0.18	-0.56
MAML3 K485Q	NP_061187	0.911	-0.268	1.29	0.23
MAP1LC3B Y113C	NP_073729	0.287	-7.928	0.20	-1.02
MLL3 G315C	NP_733751	1.000	-6.503	-0.51	-0.76
MUC16 G13386E	NP_078966	1.000	-3.704	-0.19	-0.47
MUC5B R1097H	NP_002449	0.098	3.567	-1.28	-1.28
MUC6 V1537I	NP_005952	0.001	-0.304	-0.16	-0.08
MUC6 P1539T	NP_005952	0.001	-0.820	-0.90	-0.99
MUC6 P1569L	NP_005952	0.000	-1.784	-0.30	-0.45
MUC6 L1673V	NP_005952	0.000	0.144	0.50	-0.54
MUC6 R1737P	NP_005952	0.018	1.144	-0.99	-0.43
MUC6 Q2073P	NP_005952	0.001	0.095	0.06	-0.00
MUC6 A2081G	NP_005952	0.069	-0.367	-1.25	-0.76
NCOR1 Q26K	NP_006302	0.820	-1.429	-0.38	-0.30
OR8U8 S54R	NP_001013374	0.000	3.414	-0.55	-0.10
PABPC1 L126V	NP_002559	0.987	-2.627	-1.71	-1.91
PCMTD1 R335T	NP_443169	0.853	-2.098	-1.38	-0.61
PCMTD1 P342T	NP_443169	0.999	-2.973	-0.87	-0.77
PDSS1 R28L	NP_055132	0.000	-0.100	0.17	-0.28
PHGR1 G48R	NP_001139115	0.940	-4.667	0.37	0.09
PHGR1 G48D	NP_001139115	0.999	-3.333	0.50	-0.02
PLIN4 V903M	NP_001073869	0.007	0.279	-2.72	-1.15
PLXND1 A994D	NP_055918	1.000	-4.223	-0.19	-0.42
POLR3B L368F	NP_060552	0.426	-3.734	-0.07	-0.81
POTEC A119T	NP_001131143	0.005	-0.925	-1.35	-0.76
PRDM9 R842S	NP_064612	0.002	0.950	-1.63	-1.16
PRSS3 K229E	NP_031369	0.000	-0.421	-0.59	-0.24
PRSS3 W279R	NP_031369	0.000	1.174	-2.01	-1.08
PRSS3 W279X	NP_031369	NA	NA	NA	NA
RBFOX3 A8P	NP_001076044	0.769	-2.374	-0.11	-0.04
RP1L1 V1377A	NP_849188	0.010	-0.121	0.29	-0.65
SEZ6 R963P	NP_849191	0.453	-0.984	-0.51	-0.72
SLC9B1 I279M	NP_001094344	0.372	-0.683	-1.85	-1.68
SLC9B1 I287V	NP_001094344	0.001	0.041	0.28	-1.00
SPATC1 T327P	NP_001127846	0.531	-0.596	0.29	-0.08
SPIRE2 A14T	NP_115827	0.394	-0.084	-0.61	-0.49
SSC5D H1300Y	NP_001138422	0.659	-0.277	-0.29	0.13
SSC5D M1303I	NP_001138422	0.000	-0.552	0.36	0.11

SSC5D Y1312D	NP_001138422	0.000	-0.217	-0.18	-0.54
TAS2R30 A227V	NP_001091112	0.709	-3.305	0.06	0.20
TAS2R30 L243V	NP_001091112	0.329	-2.327	-0.62	-1.38
TCHH E493Q	NP_009044	0.002	0.665	-0.06	-0.20
TMEM200B S84F	NP_001003682	0.026	-2.399	0.79	0.26
TTLL4 T467P	NP_055455	0.392	-0.452	-1.15	-0.55
TUBA3D C347F	NP_525125	0.980	-8.423	-0.08	-0.01
UBXN11 C479G	NP_663320	0.000	-0.117	-0.96	-0.63
USP6 I67M	NP_004496	0.932	0.228	-0.61	-1.26
USP6 R68W	NP_004496	0.997	-0.440	-0.58	-0.37
ZNF208 V325F	NP_009084	0.000	0.293	-2.00	-1.04
ZNF676 G261A	NP_001001411	0.004	3.582	-1.02	-1.05
ZNF717 A93T	NP_001121695	0.743	0.008	-1.15	-0.71
ZNF717 T186I	NP_001121695	0.068	-0.782	-1.09	-0.39
ZNF717 G485R	NP_001121695	1.000	-6.280	-0.85	-0.41
ZNF717 K491R	NP_001121695	0.196	-1.625	-0.16	-0.18
ZNF717 S492L	NP_001121695	0.997	-4.130	-0.63	0.31
ZNF717 R517C	NP_001121695	0.000	-0.709	-0.62	-0.81
ZNF717 F913C	NP_001121695	0.991	-0.409	-2.02	-1.61
ZNF880 K316R	NP_001138906	0.078	1.176	0.17	-0.14
ZNF880 S319T	NP_001138906	0.197	2.869	-0.03	0.09
ZXDB E122K	NP_009088	0.355	-0.721	-0.13	-0.52
